# Delleman Oorthuys syndrome: ‘Oculocerebrocutaneous syndrome’

**DOI:** 10.4103/0301-4738.55074

**Published:** 2009

**Authors:** Vipul Arora, Usha R Kim, Hadi M Khazei

**Affiliations:** Orbit, Oculoplasty and Oncology Clinic, Aravind Eye Hospital and Postgraduate Institute of Ophthalmology, Madurai, Tamil Nadu, India

**Keywords:** Cystic eye, Delleman syndrome, neurological symptoms.

## Abstract

Delleman Oorthuys syndrome (oculocerebrocutaneous syndrome) is a rare, congenital sporadic disorder affecting the skin and central nervous system. We present the case of a one-month-old male who presented with an orbital cyst in the left eye since birth along with other manifestations of this syndrome. The manifestations of this syndrome resemble other developmental disorders like Goldenhar and Goltz syndrome. Conservative management of the orbital cyst in these cases have been described. The need to diagnose this rare congenital anomaly with cerebral malformations as a separate entity is crucial in the management of these children.

The syndrome of orbital cyst and anophthalmia or microphthalmia, periocular cystic appendages, skin tags, cerebral cysts with other cerebral anomalies and focal dermal hypoplasia was first described by Delleman *et al*. in 1981.[1–4] This entity, known as oculocerebrocutaneous syndrome, is also named after those who identified it. Since it was first identified, several sporadic manifestations of this syndrome have been reported. A brief report of our case with characteristic manifestations and management is presented.

## Case Report

**Figure 1:**
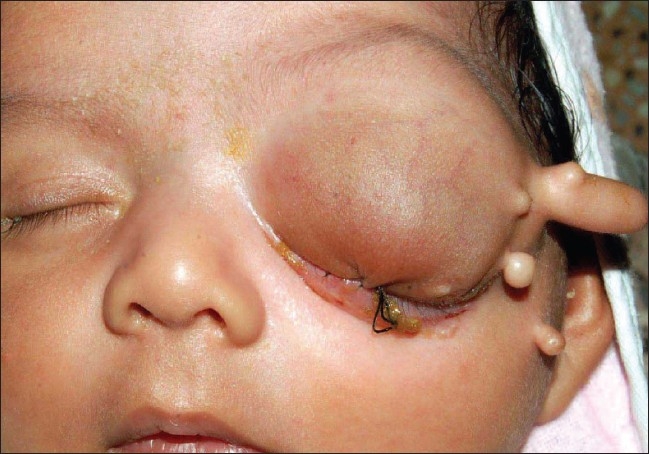
Clinical photograph of patient at one-month with congential cystic mass replacing the eyeball on left side with accessory periocular cystic appendages.

A one-month-old infant of healthy parents with a 39-year-old father and 26-year-old mother presented to us with left-sided ocular swelling which was first noticed at birth and had increased to the presenting size in one month [Fig. 1]. The child had a normal birth history. There was no history of consanguinity in the parents. There was no history of seizures. The child had one elder healthy sibling, male, aged six years.

A large cystic swelling in the child's left orbit was positive for transillumination test. Skin over the swelling was hypoplastic with prominent veins. There was no other area of skin hypoplasia elsewhere in the body. Multiple cystic appendages were present in the periorbital region. No globe was appreciated on the left side. Examination of the right eye was normal. B-scan ultrasonography of the left orbit showed fluid-filled cystic areas with no evidence of globe.

Systemic evaluation including neurological examination revealed no abnormality. Developmental milestones were normal for age.

**Figure 2:**
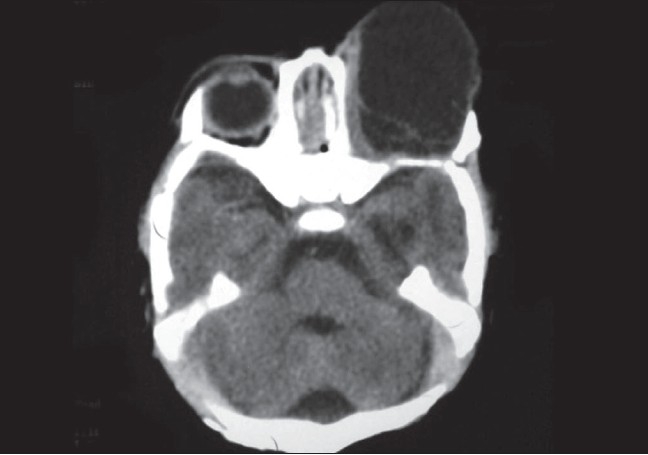
Axial CT scan of orbit showing hypodense cystic area on left side with anophthalmia

**Figure 3:**
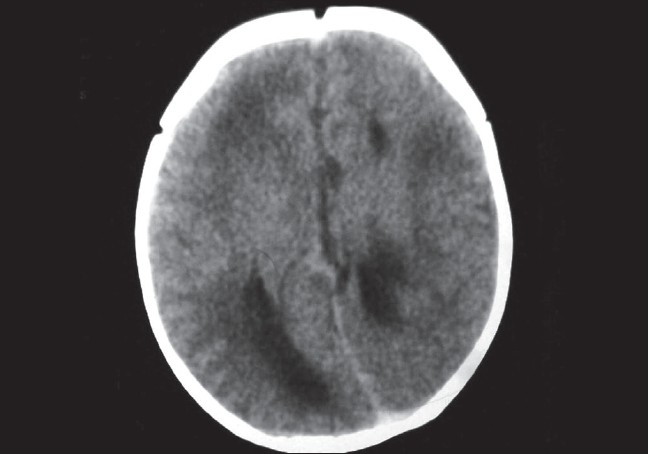
Axial CT scan of ventricles showing dilatation and separation of lateral ventricles with corpus callosum agenesis with colpocephaly

On axial computed tomographic scan there was anophthalmia with a hypodense cystic area in the left orbit. This area had no intracranial communication [Fig. 2]. There was dilatation and separation of lateral ventricles with corpus callosum agenesis, with higher placed third ventricle with colpocephaly (focal ballooning of trigone and occipital horns of lateral ventricle). However there was no evidence of hydrocephalus [Fig. 3].

**Figure 4:**
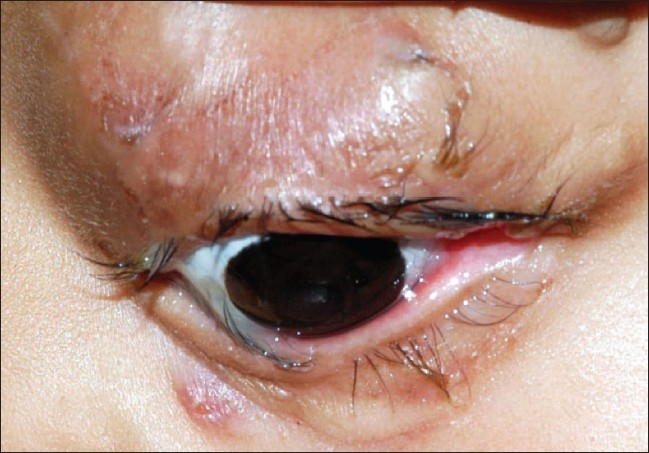
Clinical photograph of patient at one-year follow-up showing left eye with prosthesis

The patient underwent a simple cyst aspiration (with 20 mL of fluid drained). There was a recurrence of cystic swelling at one month follow-up, and repeat cyst aspiration was done with addition of ethanolamine oleate sclerotherapy (0.8 mL). Excision of periocular skin appendages was done simultaneously. This was followed by placement of a conformer in the empty socket. No recurrence of cyst was noticed in one year of follow-up [Fig. 4].

## Discussion

Oculocerebrocutaneous syndrome (OCC), a rare genetic disorder is characterized primarily by an orbital cyst with periorbital skin appendages which may be associated with anopthalmia or microphthalmia, major cerebral malformation, and focal dermal hypoplasia or aplasia.[1–4] The orbital cyst may sometimes be sufficiently large to obscure the micropthalmic eye.

Neurological abnormalities associated with OCC syndrome may include malformations of the ventricular system in the brain like colpocephaly, multiple fluid-filled cystic cavities within the cerebral and cerebellar cortex, agenesis of corpus callosum, and hydrocephalus.[1–4] Affected infants and children may demonstrate psychomotor retardation, seizures, and developmental delay. In our case, the patient did not have a history of seizures, and developmental milestones were normal for age. However, in all these cases a long-term follow-up is important to evaluate the patient's neurological function over time.

The cutaneous features include underdevelopment or absence of skin in certain localized regions (focal hypoplasia or aplasia). Characteristic of this syndrome are pink-colored or flesh-colored outgrowths of skin (cutaneous tag) within certain facial areas, most commonly the periocular area.

Lesions in the syndrome correspond to a disruption of embryonic development at a very early stage. Microphthalmos with cyst is related to incomplete closure of the embryonic fissure, normally occurring at five to six weeks,[5] with cystic extension of neuroretinal tissue through the defect into the orbit.[6] The distribution of accessory facial skin tags along the line of the fusion of facial buds suggests a similar problem with closure of these embryonic furrows.[5] Likewise, the agenesis of corpus callosum may be considered as a failure of commissural fibers to link up in the midline.

Most of the cases reported were sporadic with no reports of affected siblings. The inheritance pattern of OCC is variable, but the most common hypothesis put forward for this syndrome is mosaicism.[7] However, the continuity and symmetry of lesions makes this uncertain. It could also be the result of autosomal dominant inheritance with variable expression. Consanguinity of phenotypically normal parents in many reports (autosomal recessive inheritance) makes it more complex. In our case parents had non-consanguineous marriage, which suggests that a new mutation may be responsible.

Delleman Oorthuys syndrome shows overlapping clinical features with Goldenhar syndrome and Goltz syndrome. However, in the Goldenhar syndrome, the characteristic feature is of epibulbar dermoid and skin tags in the preauricular region with vertebral anomalies and absence of cerebral cyst. Goltz syndrome is an X-linked dominant syndrome, occurring only in females (as it is lethal in males), may have microphthalmos and coloboma and focal dermal hypoplasia but the other characteristic feature is of polysyndactyly and poor dendition.[8]

Management of orbital cyst in previously reported cases was by aspiration of cyst followed by dissection and removal of cyst from surrounding periocular structures.[4] We used sclerosing agent ethanolamine oleate in our case. The use of the agent for orbitopalpebral cyst has been well documented[9] but its use for orbital cysts in Delleman Oorthuys syndrome has not been previously reported. Surgical removal of the cyst involves prolonged anesthesia and extensive dissection. Owing to the cerebral malformations and seizures associated with this syndrome, prolonged anesthesia can be difficult.[10] We, therefore, recommend the use of a sclerosing agent for conservative management of orbital cysts.

We also recommend a detailed neurological workup in these children; and if a cerebral or cerebellar manifestation such as hydrocephalus is present, it warrants urgent neurological opinion for surgery to prevent further neurological damage. Patients with congential cystic eye with periocular skin appendages with cerebral malformation should be assessed by the ophthalmologist, neurophysician and pediatrician in close coordination for proper management of these patients.
